# Plasmonic metasurfaces manipulating the two spin components from spin–orbit interactions of light with lattice field generations

**DOI:** 10.1515/nanoph-2021-0567

**Published:** 2021-12-13

**Authors:** Ruirui Zhang, Manna Gu, Rui Sun, Xiangyu Zeng, Yuqin Zhang, Yu Zhang, Chen Cheng, Zijun Zhan, Chao Chen, Xiaorong Ren, Changwei He, Chunxiang Liu, Chuanfu Cheng

**Affiliations:** College of Physics and Electronics, Shandong Normal University, Jinan, 250014, China; School of Computer Science and Technology, Shandong University of Finance and Economics, Jinan, 250014, China; School of Science, Shandong Jianzhu University, Jinan, 250101, China; School of Electronic and Information Engineering, Qilu University of Technology (Shandong Academy of Sciences), Jinan, 250353, China

**Keywords:** metasurfaces, opticallattice fields, Pancharatnam–Berry phase, spin components, spin-orbit interactions

## Abstract

Artificial nanostructures in metasurfaces induce strong spin–orbit interactions (SOIs), by which incident circularly polarized light can be transformed into two opposite spin components. The component with an opposite helicity to the incident light acquires a geometric phase and is used to realize the versatile functions of the metasurfaces; however, the other component, with an identical helicity, is often neglected as a diffused background. Here, by simultaneously manipulating the two spin components originating from the SOI in plasmonic metasurfaces, independent wavefields in the primary and converted spin channels are achieved; the wavefield in the primary channel is controlled by tailoring the dynamic phase, and that in the converted channel is regulated by designing the Pancharatnam–Berry phase in concurrence with the dynamic phase. The scheme is realized by generating optical lattice fields with different topologies in two spin channels, with the metasurfaces composed of metal nanoslits within six round-apertures mimicking the multi-beam interference. This study demonstrates independent optical fields in a dual-spin channel based on the SOI effect in the metasurface, which provides a higher polarization degree of freedom to modify optical properties at the subwavelength scale.

## 1 Introduction

Photonic spin–orbit interactions (SOIs) are optical phenomena in which the spin affects the spatial distribution and propagation of light [1]; it is related to the geometric phase and interaction between the spin angular momentum (SAM) and orbital angular momentum (OAM). Recently, SOI has gained significant interest in the exploration and understanding of a variety of unusual physical effects. The optical spin Hall effect [2], [3], [4] is a fundamental phenomenon, originating from SOI, which exhibits a spin-dependent transverse beam shift at the medium interface or through inhomogeneous media. Another important effect of SOI is spin-to-orbit conversions (SOC) [5], which is realized in the focusing of nonparaxial beams and scattering from small particles [6]. Metamaterials and metasurfaces, as inhomogeneous and anisotropic media, also demonstrate strong SOI effects, and provide powerful spin-dependent control of wavefronts [7, 8] and easier implementation of SOC. More interestingly, in the surface mode of evanescent waves, the optical quantum spin Hall effect [9] appears and exhibits strong spin-momentum locking, which originates from the transverse spin locked to a unidirectional propagation, and is similar to the surface state of the topological insulator. Overall, the effects in SOIs have offered numerous important applications in broad areas, such as nanodevice design [10], precision metrology [11], topological insulators [12], and quantum information processing [13, 14].

As a paradigm of the enhanced SOI effect, metasurfaces are planar interfaces composed of artificial nanostructures with various dimensions and orientations [15, 16]; they regulate the amplitude, phase, and polarization of the output light via the interaction between the SAM and nanostructures [1]. Owing to their extraordinary capabilities in reshaping optical wavefronts into desired profiles, metasurfaces have been applied to metalenses [17, 18], holography [19], [20], [21], vortex generators [22, 23], and polarization controllers [24], [25], [26]. An incident circularly polarized beam, of helicity *σ* (*σ* = ±1) for left and right circular polarization (LCP and RCP), transmitted through the metasurface is decomposed into two components [27]. One component has the same helicity as the incident light, referred to as the primary spin component, and the other has the opposite helicity with the PB phase Φ*g* = 2*σθ*, referred to as the converted spin component, which is the field to be manipulated in most common metasurface design. The converted spin components under the incident helicity ±*σ* are conjugate, owing to the opposite PB phase. The combination of the dynamic phase and PB phase is a novel and potent SOI scheme for helicity-switchable metasurfaces, which enables the imposition of independent phases on the converted component under reversal of incident helicity *σ*, realizing an independent control of the output field. This scheme has notably extended the applications of metasurfaces to spin selective holography [28, 29] and vector holography [30], vector beam generation with SOC [22, 31], full-Stokes polarization camera design [32], and achromatic imaging and lensing [33, 34].

Produced by the interference of multiple laser beams, the photonic lattice fields have periodic intensity and polarization patterns, and they were originally used as the periodic potentials to form artificial cold atom lattices [35]. The forms of the lattice fields depend on the intersection geometry and the phase configuration of the beams, and the familiar types of optical lattice fields of different orders include the honeycomb, Kagome, and hexagon vortex lattices [36]. In recent years, the creation of photonic lattice fields has become an interesting novel topic, and the fields can be translated into materialized lattices, which have enabled the newly emerged field of topological photonics [37, 38] and other areas of research [39, 40]. Exemplarily, the lattice fields enable the construction of pseudospin states [41] with different degrees of freedom, such as OAM [42], polarization [43], and crystal lattice symmetry [41, 44], to realize edge states without breaking time-reversal symmetry [45]. The topology of the nanoscale lattice fields can be controlled by SOI in metasurfaces and nanoslit polygons based on either the PB phase or its combination with the dynamic phase. With the PB phase metasurfaces, topology is achieved for the hexagonal vortex lattice through the quasi-Talbot effect [46], and the Kagome lattice through an additional prism deflection phase [47]. With the combination of the PB phase and dynamic phase, control over lattice topology is realized with more flexibility. By arraying metasurface nanostructures on truncated spirals, all four lattices, including the two other forms of lattices (namely, hexagonal and honeycomb), are acquired [48]. By repositioning a slit side or diagonal pair of slit sides of a hexagon to modulate the dynamic phase, Tsesses et al. realized the photonic skyrmion lattice and its transition from bubble-type to Néel-type [49], and have regulated different lattice topologies [50].

In the previous studies for generating lattice fields, only the converted spin component is considered, and two different fields of this component are obtained under the corresponding illuminations of the two spin helicities [46], [47], [48, 50], whereas the primary spin component has been typically neglected and scattered uncontrollably as futile background noise. With the dynamic phase to directly control field of the primary component, and the PB phase cooperatively to control the converted component, this work realizes the independent output of two spin fields under certain incident spin helicity, making a novel contribution to the literature by utilizing the primary spin component as a new channel for generating desired field. For implementation demonstration, the metasurfaces are designed with nanoslits distributed within six round apertures in the analogy of multi-beam interference to generate hexagon-based lattices, enabling lattice creation in the two spin channels. The dynamic phase is introduced by a spiral, on which the centers of the round-apertures are located, and the lattice topology in the primary spin channel is regulated into the desired form by adjusting the pitch of the spiral. Considering its combination with the dynamic phase, the PB phase from the varied orientation of the nanoslit in each round-aperture is designed accordingly to provide a phase shift between adjacent beams, and the lattice topology of the converted spin channel is controlled. Furthermore, when incident helicity *σ* is reversed to −*σ*, a different lattice of the converted component is acquired, demonstrating a helicity-dependent characteristic of the lattice topology, while the lattice topology of the primary component remains unchanged. The lattice fields of the two components exist in two respective spin channels, which overlap spatially, and they are experimentally acquired by a polarization filter (PF) composed of a quarter-wave plate and polarizer. The control over the dual-spin-channel lattices demonstrates that the SOI effect in metasurfaces provides the polarization degree of freedom for the manipulation of light field at the nanoscale, which is of significance to a variety of applications, in areas such as integrated photonic systems [51], topological photonics [52, 53], and ultra-thin quantum metadevices [54, 55].

## 2 Principle analysis and structure design

**Figure 1:**
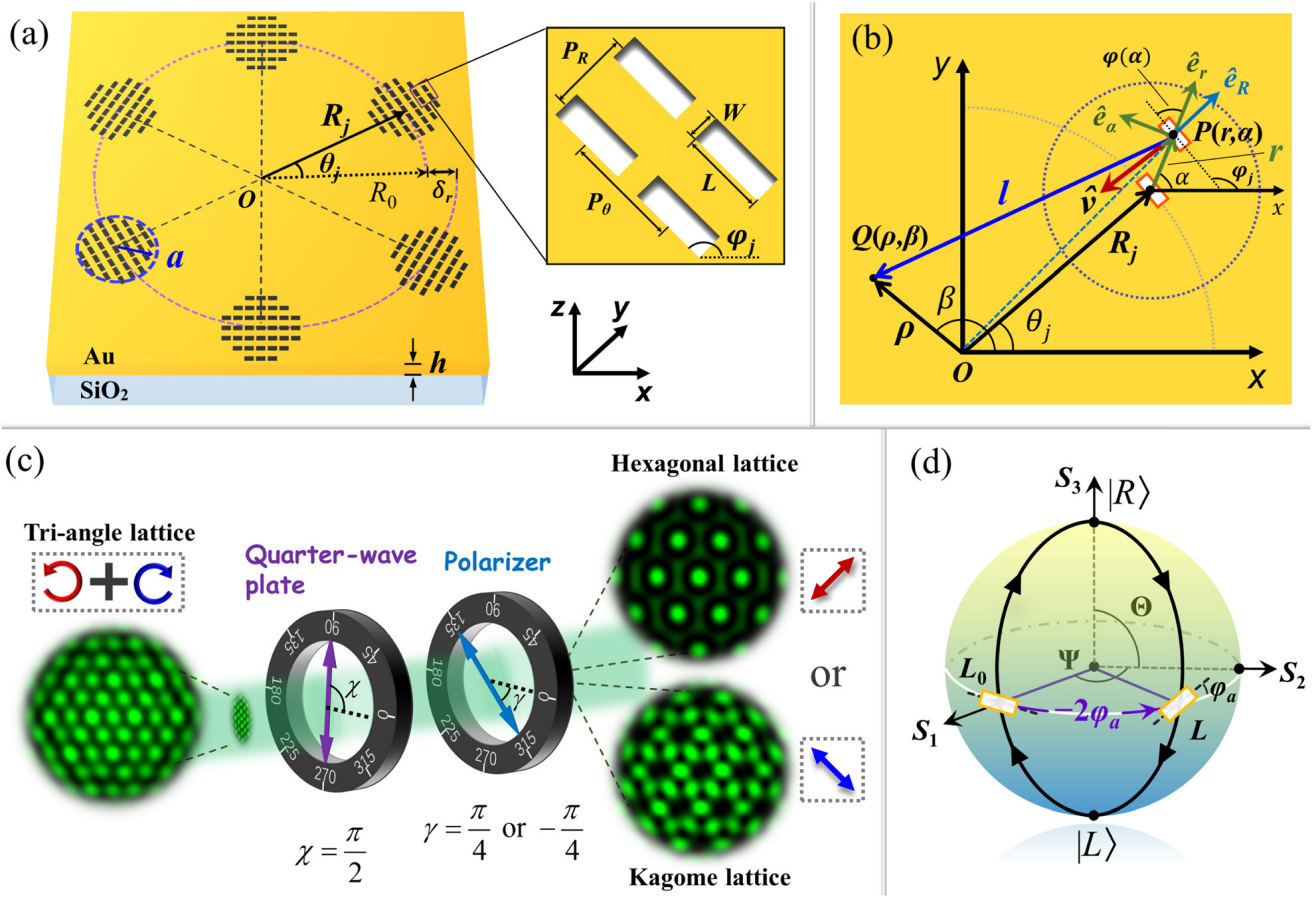
Demonstration of principle and method for metasuface designs (a) Schematic of the metasurface. The radius of each aperture is *a* = 1.75 μm, the spiral at which the apertures are located has a primary radius *R*_0_ = 12 μm, and the thickness of the gold film is *h* = 200 nm. The nanoslits are arranged in a square lattice with lattice constant *P*_*R*_ = *P*_*θ*_ = *λ*_spp_. The length of the slit *L* = 250 nm and width *W* = 110 nm. (b) Illustration of the slit orientation and local polar coordinates. (c) Illustration of the polarization filtering processing. *χ* and *γ* denote the fast axis and transmission axis directions of the QWP and polarizer, respectively. (d) Poincaré sphere for illustration of the conversion of the polarization state and geometric phase change of the converted spin component.

The metasurface is designed, based on the SOI effect, to manipulate the light field into two channels – of primary and converted spin – with the generation of optical lattice fields used as a practical demonstration; Figure 1(a) shows a schematic of the metasurface. *N* round apertures, composed of nanoslits with identical dimensions, lie on a spiral depicted by *R* = *R*_0_ + *mλ*_spp_*θ*/2*π* in polar coordinates (*R*, *θ*), where *R*_0_ is the primary radius of the spiral, *λ*_spp_ is the surface plasmon polariton (SPP) wavelength, and *δ*_*r*_ = *mλ*_spp_ describes the spiral pitch with integer *m* denoting the geometric order of the spiral. The azimuthal and radial coordinates of the center of the *j*th aperture are *θ*_*j*_ = (2*j* + 1)*π*/*N* and *R*_*j*_ = *R*_0_ + *δ*_*r*_*θ*_*j*_/2*π*, respectively, with *j* being an integer from 0 to *N* − 1, and the position vector of the central-slit is ***R***_***j***_ (*R*_***j***_, *θ*_*j*_). The metasurface is intuitively designed to contain *N* = 6 apertures at the vertices of a hexagon. While the arrangement of the apertures provides the geometry for multi-beam-interference, the SOI of the nanoslits with the incident light will create two spin channels and provide different phase fronts for the expected wavefields.

We now analyze the wave fields launched by a single nanoslit of the metasurface under incident circular polarization. As illustrated in Figure 1(b), within the *j*th aperture, a nanoslit is located at *P*(*r*, *α*) with position vector ***r*** in local polar coordinates (*r*, *α*) and the origin at the aperture center (*R*_*j*_, *θ*_*j*_). All nanoslits in the aperture have identical orientation angles *φ*_*j*_ = *α* + *φ*(*α*), where *φ*_*j*_ is a constant defined as the angle between the slit and *x*-axis in the local coordinate system, and *φ*(*α*) is the angle between the slit and radial unit vector ${\hat{e}}_{r}$. It is well understood that the slit acts as a local subwavelength linear polarizer (LP), of which the transmitting direction is perpendicular to its longitudinal axis, and it is determined by the unit vector $\hat{v}=-\text{sin}\left[\varphi \left(\alpha \right)\right]{\hat{e}}_{r}+\text{cos}\left[\varphi \left(\alpha \right)\right]{\hat{e}}_{\alpha }$. The incident plane wave ***E***_in_ of circular polarization illuminating the metasurface can be expressed in the local polar coordinates (*r*, *α*) as

(1) $$E_{\text{in}}=\frac{1}{\sqrt{2}}{\text{e}}^{i\sigma \alpha }\left({\hat{e}}_{r}+i\sigma {\hat{e}}_{\alpha }\right)$$

Using the expression of the unit vector $\hat{v}$ and ***E***_in_, we can write the SPP field ***E***_*j*_ (*r*, *α*; *σ*) launched by the nanoslit in linear polarization as [56]

(2) $$E_{j}\left(r\text{, }\alpha \text{; }\sigma \right)=\left(E_{in}\cdot \hat{v}\right)\hat{v}=\frac{i\sigma }{\sqrt{2}}{\text{e}}^{i\sigma \varphi_{j}}\left[\begin{array}{l} \text{sin}\left(\alpha -\varphi_{j}\right)\\ \text{cos}\left(\alpha -\varphi_{j}\right) \end{array}\right]$$

where *r* and *α*, labeled in ***E***_*j*_ (*r*, *α*; *σ*) on the left side of the equation, indicates that the SPP field from the nanoslit is described in the local polar coordinate system (*r*, *α*). Notably, the incident circular polarization ***E***_in_, given by Eq. (1), transforming into the linear polarization of the outgoing wavefield ***E***_*j*_ (*r*, *α*; *σ*) with a phase shift, may enable control over the propagation and distribution of light, exhibiting a strong SOI effect.

To investigate the light field at observation point *Q*(*ρ*, *β*) near the center of the metasurface, we assume a global polar coordinate system (*ρ*, *β*) with unit vector (${\hat{e}}_{\rho },{\hat{e}}_{\beta }$), as shown in Figure 1(b), and express the wavefield ***E***_*j*_ (*r*, *α*; *σ*) in Jones vectors in this system, denoted by ***E***_*j*_ (${\hat{e}}_{\rho },{\hat{e}}_{\beta }$; *r*, *α*; *σ*). By transforming the local unit vectors (${\hat{e}}_{r},{\hat{e}}_{\alpha }$) into (${\hat{e}}_{\rho },{\hat{e}}_{\beta }$) with a rotation of angle (*β* − *α*), the wavefield outgoing from the slit is then written as

(3) $$E_{j}\left({\hat{e}}_{\rho }\text{, }{\hat{e}}_{\beta }\text{; }r\text{, }\alpha \text{; }\sigma \right)=\left[\begin{array}{l} \text{cos}\left(\beta -\alpha \right)\text{sin}\left(\beta -\alpha \right)\\ -\text{sin}\left(\beta -\alpha \right)\text{cos}\left(\beta -\alpha \right) \end{array}\right]E_{j}\left(r\text{, }\alpha \text{; }\sigma \right)$$

The single slit at point *P*(*r*, *α*) can be regarded as a secondary source contributing to the wavefield ***E*** (*ρ*, *β*; *σ*) at point *Q*(*ρ*, *β*), with *σ* again representing the helicity of the illuminating light. Then, ***E*** (*ρ*, *β*; *σ*) represents the interference of the wavelets from all the slits in the metasurface, and it can be calculated as

(4) $$E\left(\rho \text{, }\beta \text{; }\sigma \right)=\sum_{j=0}^{N-1}\int_{0}^{2\pi }\int_{0}^{a}E_{j}\left({\hat{e}}_{p}\text{, }{\hat{e}}_{\beta }\text{; }r\text{, }\alpha \text{; }\sigma \right){\text{e}}^{ik_{\text{sp}}\cdot I}r\text{d}r\text{d}\alpha$$

where ***k***_sp_ is the wave vector of the wave propagating from the slit to point *Q*(*ρ*, *β*). Analogously, *ρ* and *β*, labeled in ***E*** (*ρ*, *β*; *σ*) on the left side of Eq. (4), represent the case of the wavefield being expressed in the global polar coordinate system (*ρ*, *β*). Equation (4) considers that the slits in each aperture are uniformly distributed, and the superposition of the wavelets has been changed from discrete slits to the integral over the area in the aperture. In the calculation of the position vector ***l*** = ***ρ*** − ***R***_*j*_ − ***r*** of point *P* relative to point *Q*, both the radius *a* of the aperture and radial coordinate *ρ* are significantly smaller than the radius *R*_*j*_, i.e., *a* ≪ *R*_*j*_ and *ρ* ≪ *R*_*j*_. Then, the phase retardation in Eq. (4) is approximated as ***k***_sp_⸱ ***l*** ≈ 2*π*[*R*_*j*_ + *r* cos(*α* − *θ*_*j*_) − *ρ* cos(*β* − *θ*_*j*_)]/*λ*_spp_. Note that *φ*_*j*_ = *α* + *φ*(*α*) reduces to *φ*_*j*_ = *θ*_*j*_ + *π*/2 from the geometry shown in Figure 1(b). Considering the variation of *R*_*j*_ with *θ*_*j*_, and substituting Eqs. (2) and (3) into Eq. (4), we obtain

(5) $$\begin{array}{l} E\left(\rho \text{, }\beta ;\sigma \right)=\frac{{\text{e}}^{ik_{\text{sp}}R_{0}}}{\sqrt{2}}\sum_{j=0}^{N-1}{\text{e}}^{i\left[\left(m+\sigma \right)\theta_{j}-k_{\text{sp}}\rho \text{ cos}\left(\beta -\theta_{j}\right)\right]}\left[\begin{array}{l} \text{cos}\left(\beta -\theta_{j}\right)\\ -\text{sin}\left(\beta -\theta_{j}\right) \end{array}\right]\\ \times \sum_{k=-\infty }^{+\infty }{\left(i\right)}^{k}{\text{e}}^{ik\theta_{j}}\int_{0}^{a}rJ_{k}\left(k_{\text{sp}}r\right)\text{d}r\int_{0}^{2\pi }{\text{e}}^{-ik\alpha }\text{d}\alpha \end{array}$$

where the generating function exp[*ikr* cos(*α* − *θ*_*j*_)] of the integer-order Bessel function is expanded, and ***J***_*k*_(*k*_sp_*r*) represents the first type of Bessel function with order *k*. The integrals inside the second summation in Eq. (5) vanish if *k* ≠ 0, and ***E*** (*ρ*, *β*; *σ*) is derived as

(6) $$E\left(\rho \text{, }\beta ;\sigma \right)=E_{L}\left(\rho \text{, }\beta ;\sigma \right)|L_{\rho \beta }〉+E_{R}\left(\rho \text{, }\beta ;\sigma \right)|R_{\rho \beta }〉$$

with

(7) $$\left\{ \begin{array}{l} E_{L}\left(\rho \text{, }\beta ;\sigma \right)=C\sum_{j=0}^{N-1}{\text{e}}^{i\left(\sigma +m-1\right)\theta_{j}}{\text{e}}^{-ik_{\text{sp}}\rho \text{ cos}\left(\beta -\theta_{j}\right)}\\ E_{R}\left(\rho \text{, }\beta ;\sigma \right)=C\sum_{j=0}^{N-1}{\text{e}}^{i\left(\sigma +m+1\right)\theta_{j}}{\text{e}}^{-ik_{\text{sp}}\rho \text{ cos}\left(\beta -\theta_{j}\right)} \end{array}\right.$$

where $C=\pi aJ_{1}\left(k_{\text{sp}}a\right)\text{exp}\left(ik_{\text{sp}}R_{0}\right)/\sqrt{2}k_{\text{sp}}$, and $|L_{\rho \beta }〉=\exp\left(i\beta \right){\left[1i\right]}^{\text{T}}$ and $|R_{\rho \beta }〉=\exp\left(-i\beta \right){\left[1-i\right]}^{\text{T}}$ represent the LCP and RCP in (*ρ*, *β*) coordinates, respectively. Equations (6) and (7) show that ***E*** (*ρ*, *β*; *σ*) includes spin components $E_{L}\left(\rho ,\beta ;\sigma \right)|L_{\rho \beta }〉$ for LCP and $E_{R}\left(\rho ,\beta ;\sigma \right)|R_{\rho \beta }〉$ for RCP. For the incident light of the LCP, the first term $E_{L}\left(\rho ,\beta ;\sigma \right)|L_{\rho \beta }〉$ has the same helicity as that of the incident waves, which can be referred to as the primary spin components of the wavefields, and the second term $E_{R}\left(\rho ,\beta ;\sigma \right)|R_{\rho \beta }〉$ is referred to as the converted spin component with a helicity opposite to that of the incident waves. For the incident light of the RCP, the terms $E_{R}\left(\rho ,\beta ;\sigma \right)|R_{\rho \beta }〉$ and $E_{L}\left(\rho ,\beta ;\sigma \right)|L_{\rho \beta }〉$ are the primary and converted spin components, respectively. Alternatively, the LCP or RCP component with the same helicity as the incident light is the primary spin component, and the other, with the opposite helicity, is the converted spin component. In the above derivations, we see that the spiral radius *R*_*j*_ = *R*_0_ + *mλ*_spp_*θ*_*j*_/2*π* in the optical path | ***l*** | contributes to the dynamic phase factor exp(*i* ***k***_sp_⸱ ***l***), and provide the constant phase *mθ*_*j*_ for waves from the nanoslits in the *j*th aperture, and then it independently or incorporating with geometric phase sets the constant phase increment for waves from two adjacent apertures to produce the lattice field of certain order.

The spatial distributions of the component fields *E*_*L*_(*ρ*, *β*; *σ*) and *E*_*R*_(*ρ*, *β*; *σ*) are dependent on two factors: one is the geometry of the arrangement of nanoslits in the metasurface, appearing as the phase $\text{exp}\left[-ik_{\text{sp}}\rho \text{ cos}\left(\beta -\theta_{j}\right)\right]$, and the other is the phase profiles of $\text{exp}\left[i\left(\sigma +m-1\right)\theta_{j}\right]$ and $\text{exp}\left[i\left(\sigma +m+1\right)\theta_{j}\right]$ designed for the two components, respectively. Although these two factors take effect concurrently in manipulating a general light field with a metasurface, in generating the exemplary lattice fields, the roles of the two factors are more significant. The former compares the geometry of the plane wave beams to determine the lattice structures, such as the hexagon types of the lattices for the six-aperture metasurface, and the latter represents the phase profiles imparted to the analog beams to determine the detailed lattice topologies, such as Kagome and honeycomb, of the primary spin and converted spin components. Furthermore, in the case of LCP (*σ* = 1) incidence, the primary spin component *E*_*L*_(*ρ*, *β*; *σ*), with phase profile $\text{exp}\left[i\left(\sigma +m-1\right)\theta_{j}\right]=\text{exp}\left[im\theta_{j}\right]$, is completely determined by the dynamic phase *mθ*_*j*_ from the optical path difference, related to the radius change *R*_*j*_ of the spiral over *θ*_*j*_. Meanwhile, the converted spin component *E*_*R*_(*ρ*, *β*; *σ*), with the corresponding phase profile $\text{exp}\left[i\left(\sigma +m+1\right)\theta_{j}\right]=\text{exp}\left[i\left(m+2\sigma \right)\theta_{j}\right]$, depends on the combination of the dynamic phase *mθ*_*j*_ and PB phase 2*σθ*_*j*_, related to the orientation of the slits in the *j*th aperture. Notably, when the incident waves are switched from LCP to RCP (*σ* = −1), the wavefield of the primary spin component remains unchanged; however, the converted spin component changes owing to the shift of the phase factor from $\text{exp}\left[i\left(m+2\right)\theta_{j}\right]$ to $\text{exp}\left[i\left(m-2\right)\theta_{j}\right]$ with the reversal of incident helicity, leading to different lattices of the converted spin component. This shift is attributed to the asymmetry of factors $\text{exp}\left[i\left(\sigma +m-1\right)\theta_{j}\right]$ or $\text{exp}\left[i\left(\sigma +m+1\right)\theta_{j}\right]$ in Eq. (7) with respect to *σ*, which results from the introduction of the dynamic phase *mθ*_*j*_ from the spiral, and it is equivalent to the asymmetric SOI effect [57] in the metasurface. More intuitively, the conversion of the incident light to the converted spin component can be schematically demonstrated on the Poincaré sphere [58, 59], as shown in Figure 1(d). For the incident light of RCP at north pole, the converted spin component of LCP in the wavefield transmitting through a nanoslit of orientation angle *φ* is at the south pole, with nanoslit acting equivalently as a half-wave plate with axis orientation angle *φ*_*a*_ = *φ* − *π*/2, and the corresponding path is (RCP) → *L* (*φ*_*a*_ = *φ* − *π*/2) → (LCP). The path (LCP) → *L*_0_ (*φ*_*a*_ = 0) → (RCP) for the nanoslit with *φ*_*a*_ = 0 to convert the incident LCP to RCP is taken as the reference path, and the two paths form the enclosed path. From the solid angle suspended by the enclosed path to sphere center, the geometric phase 2*σφ*_*a*_ (here *σ* = −1) is obtained; and particularly, when *φ*_*a*_ = *θ*_*j*_, the geometric phase is 2*σθ*_*j*_. While for the field of primary spin component, the state of circular polarization is not changed and it stays at the North Pole, and no geometric phase is imposed.

The wavefields of the primary and converted spin components can be manipulated in the two independent spin channels; however, they are spatially overlapped and indistinguishable, resulting in a mixed spatial output. As shown in Figure 1(c), we propose a separation of the two component fields in the circular polarization filtering system, which consists of a quarter-wave plate (QWP) and LP. In the global polar coordinate system (*ρ*, *β*), the operation of the QWP on a light field is expressed by its Jones matrix ${\bar{T}}_{\text{qw}}\left(\chi \text{; }\beta \right)$:

(8) $${\bar{T}}_{\text{qw}}\left(\chi \text{; }\beta \right)=\left[\begin{array}{l} {\text{cos}}^{2}\left(\chi -\beta \right)+i{\text{sin}}^{2}\left(\chi -\beta \right)\left(1-i\right)\text{sin}\left(\chi -\beta \right)\text{cos}\left(\chi -\beta \right)\\ \left(1-i\right)\text{sin}\left(\chi -\beta \right)\text{cos}\left(\chi -\beta \right){\text{sin}}^{2}\left(\chi -\beta \right)+i{\text{cos}}^{2}\left(\chi -\beta \right) \end{array}\right]$$

where the upper bar designates the matrix, and *χ* is the fast axis angle of the QWP with respect to the *x*-axis. The Jones matrix of the LP is given by

(9) $${\bar{T}}_{\text{lp}}\left(\gamma \text{; }\beta \right)=\frac{1}{2}\left[\begin{array}{l} 1+\text{cos}\left(2\left[\gamma -\beta \right]\right)\text{sin}\left[2\left(\gamma -\beta \right)\right]\\ \text{sin}\left[2\left(\gamma -\beta \right)\right]1-\text{cos}\left[2\left(\gamma -\beta \right)\right] \end{array}\right]$$

where *γ* is the angle of the transmitting axis of the LP with respect to the *x*-axis. In the following discussion, we set the fast axis of the QWP in the direction of the *y*-axis for substantiated analysis, i.e., *χ* = *π*/2, and the operation of the QWP on the light field ***E*** (*ρ*, *β*; *σ*) at point *Q* given by Eqs. (6) and (7) creates the wavefield ***E***_qw_(*ρ*, *β*; *σ*) = ${\bar{T}}_{\text{qw}}$(*χ* = *π*/2; *β*) ***E*** (*ρ*, *β*; *σ*). Then, the wavefield ***E***_qw_(*ρ*, *β*; *σ*) transmitted through the QWP is

(10) $$E_{\text{qw}}\left(\rho \text{, }\beta \text{; }\sigma \right)=iE_{L}\left(\rho \text{, }\beta \text{; }\sigma \right)|LP_{\rho \beta \text{,}\pi /4}〉+iE_{R}\left(\rho \text{, }\beta \text{; }\sigma \right)|LP_{\rho \beta \text{,}-\pi /4}〉$$

where the two matrices

(11) $$|LP_{\rho \beta ,\pi /4}〉=\left[\begin{array}{l} \cos\;\beta +\sin\;\beta \\ \cos\;\beta -\sin\;\beta \end{array}\right]$$

and

(12) $$|LP_{\rho \beta ,-\pi /4}〉=\left[\begin{array}{l} \cos\;\beta -\sin\;\beta \\ -\cos\;\beta -\sin\;\beta \end{array}\right]$$

are the Jones vectors of the light wave linearly polarized at *π*/4 and in −*π*/4, respectively, in the (*ρ*, *β*) coordinate system. In deriving Eq. (10), we have used ${\bar{T}}_{\text{qw}}\left(\chi =\pi /2\text{; }\beta \right)|L_{\rho \beta }〉=|LP_{\rho \beta \text{,}\pi /4}〉$ and ${\bar{T}}_{\text{qw}}\left(\chi =\pi /2\text{; }\beta \right)|R_{\rho \beta }〉=|LP_{\rho \beta \text{,}-\pi /4}〉$, indicating that the QWP transforms the wavefield of the primary and converted spin components into orthogonal linear polarizations, and they can be extracted using an LP or spatially separated by a polarizing beam splitter. Subsequent to the transmission through the LPs with the transmitting axes in *γ* = *π*/4 and −*π*/4, the wavefield at point *Q* is transformed to ***E***_PF_(*ρ*, *β*; *σ*) = ${\bar{T}}_{\text{lp}}$(*γ*; *β*) ***E***_qw_(*ρ*, *β*; *σ*). Based on Eqs. (10)–(12), ***E***_PF_(*ρ*, *β*; *σ*)|_*γ*=*π*/4_ and ***E***_PF_(*ρ*, *β*; *σ*)|_*γ*=−*π*/4_ can be written as:

(13) $$E_{\text{PF}}^{\pi /4}\left(\rho \text{, }\beta \text{; }\sigma \right)=iC\sum_{j=0}^{N-1}{\text{e}}^{il_{1}\theta_{j}}{\text{e}}^{-ik_{\text{sp}}\rho \text{ cos}\left(\beta -\theta_{j}\right)}|LP_{\rho \beta \text{,}\pi /4}〉$$

and

(14) $$E_{\text{PF}}^{-\pi /4}\left(\rho \text{, }\beta \text{; }\sigma \right)=iC\sum_{j=0}^{N-1}{\text{e}}^{il_{2}\theta_{j}}{\text{e}}^{-ik_{\text{sp}}\rho \text{ cos}\left(\beta -\theta_{j}\right)}|LP_{\rho \beta \text{,}-\pi /4}〉$$

where *l*_1_ = *σ* + *m* − 1 and *l*_2_ = *σ* + *m* + 1 are the orders of the lattices transmitted as linear polarizations $|LP_{\rho \beta \text{,}\pi /4}〉$ and $|LP_{\rho \beta \text{,}-\pi /4}〉$, respectively. Combining Eqs. (8)–(14), we observe that the PF has extracted the wavefields of the two components, by transforming $|L_{\rho \beta }〉$ into *γ* = *π*/4 linear polarization $|LP_{\rho \beta \text{,}\pi /4}〉$ and $|R_{\rho \beta }〉$ into *γ* = −*π*/4 linear polarization $|LP_{\rho \beta \text{,}-\pi /4}〉$. Notably, at the incidence of LCP, i.e., *σ* = 1, the *γ* = *π*/4 linear polarization component in Eq. (13), transformed from the LCP component *E*_*L*_(*ρ*, *β*; *σ*) of the same helicity *σ*, is the primary spin component field. Furthermore, the *γ* = −*π*/4 linear polarization component in Eq. (14), transformed from the RCP field *E*_*R*_(*ρ*, *β*; *σ*) of the opposite helicity, is the converted spin component. Similarly, with an incidence of RCP with *σ* = −1, the *γ* = *π*/4 linear polarization component in Eq. (13), from the LCP component *E*_*L*_(*ρ*, *β*; *σ*) with helicity opposite to *σ* incidence, becomes the converted spin component field. Moreover, the *γ* = −*π*/4 linear polarization component in Eq. (14), from the RCP component *E*_*R*_(*ρ*, *β*; *σ*) with the same helicity as the *σ* incidence, becomes the primary spin component field.

The different lattice topologies of the LCP and RCP components depend on the phases Φ_1*j*_ = *l*_1_*θ*_*j*_ and Φ_2*j*_ = *l*_2_*θ*_*j*_ corresponding to each aperture, if the geometry of the aperture arrangement is definite. Specifically, in the six-aperture regime of *N* = 6, the lattices include the hexagon-type topologies of hexagonal, hexagonal vortex, Kagome, and honeycomb [36], when the lattice order *l* = 0, ±1, ±2, and ±3, respectively, with *l* designating *l*_1_ and *l*_2_. For a lattice of order *l*, the wavefields of two adjacent apertures require a phase increment ΔΦ = *l*Δ*θ* = 2*lπ*/*N*, with *N* = 6 and Δ*θ* = *θ*_*j*_ − *θ*_*j*−1_ = 2*π*/*N*, or the cumulative phase difference is ΔΦ_t_ = 2*lπ* with aperture index *j* successively iterating through all the *N* apertures. Notably, the phase increments ΔΦ or ΔΦ_t_ are related to the three parameters *σ*, *m*, and the rotation of the slits. Considering the LCP incidence, the lattice order *l*_1_ of the primary spin component is directly determined by the dynamic phase ΔΦ_d_ = 2*mπ* related to the spiral pitch *mλ*_spp_, achieving *l*_1_ = *m*. In contrast, the lattice topology of the converted spin component depends on both the dynamic phase ΔΦ_d_ and geometric phase ΔΦ_g_ = 4*σπ* from the variation of the slit orientations, and the lattice order is given by *l*_2_ = 2*σ* + *m*. This indicates that the lattice topology of the primary component is independent of the spin helicity *σ* and geometrical phase, and that of the converted component is spin-dependent and spin-asymmetric.

Then, the lattice topologies are specifically related to the nanoslit arrangement in a metasurface sample. We first consider a metasurface, denoted as sample A, with the six apertures located in a circle, resulting in parameter *m* = 0. From Eq. (13), the primary spin component is the LCP wavefield filtered as $E_{\text{PF}}^{\pi /4}\left(\rho \text{, }\beta ;\sigma \right)$ under the incidence of LCP light, and its lattice is of order *l*_1_ = 0, indicating that it is a hexagonal lattice. For the converted spin component of the RCP wavefield, filtered as $E_{\text{PF}}^{-\pi /4}\left(\rho \text{, }\beta ;\sigma \right)$ in Eq. (14), have a cumulative phase difference ΔΦ_t_ = 4*π* = 2*l*_2_*π* (or phase increment ΔΦ = *l*_2_Δ*θ* = 2*π*/3), indicating lattice order of *l*_2_ = 2 appearing as a Kagome lattice. This wavefield, modulated by the geometric phase, is spin-dependent, and typically results from the SOI effect. Furthermore, the exemplary polarization filtering process of sample A under LCP incidence is shown in Figure 1(c). The unfiltered intensity pattern of the primary and converted spin components, which are spatially overlapped, presents the morphology of a triangle lattice; the hexagonal lattice, filtered as $E_{\text{PF}}^{\pi /4}\left(\rho \text{, }\beta ;\sigma \right)$, or the Kagome lattice, filtered as $E_{\text{PF}}^{-\pi /4}\left(\rho \text{, }\beta ;\sigma \right)$, can then be achieved by rotating the polarizer with its axis set to *γ* = *π*/4 or −*π*/4. When the illuminating light is changed to RCP, the helicity of the incident light is reversed to *σ* = −1, the RCP output wavefield of $E_{\text{PF}}^{-\pi /4}\left(\rho \text{, }\beta ;\sigma \right)$ in Eq. (14) is of the same helicity as the incident light (it becomes the primary spin component), and the lattice order is *l*_2_ = 0 with the topology of a hexagonal lattice. Correspondingly, the wavefield $E_{\text{PF}}^{\pi /4}\left(\rho \text{, }\beta ;\sigma \right)$ in the LCP channel becomes the converted spin component; however, the cumulative phase difference of the wavefield becomes ΔΦ_t_ = −4*π* = 2*l*_1_*π*, leading to a Kagome lattice of order *l*_1_ = −2 with a lattice vortex of the opposite topological charge. The unchanged morphology of the Kagome lattice fields of the two converted components, under the shift of the incident light from LCP and RCP, demonstrates that the metasurface is SOI-symmetric; however, from the shift, the phase fronts of the converted component lattice fields become opposite, owing to the inversed geometrical phase.

We further examine the other case of the six apertures located on a spiral of geometrical order *m* = 1, with the metasurface denoted as sample B. Under the LCP incidence with *σ* = 1, the cumulative dynamic phase ΔΦ_d_ = 2*mπ* = 2*π*, owing to the spiral pitch *mλ*_spp_, leads to the lattice order *l*_1_ = 1 for the primary spin component, which is the LCP wavefield filtered as $E_{\text{PF}}^{\pi /4}\left(\rho \text{, }\beta ;\sigma \right)$, taking the topology of a hexagonal vortex lattice. In contrast, the lattice field of the converted spin component is the RCP wavefield, filtered as $E_{\text{PF}}^{-\pi /4}\left(\rho \text{, }\beta ;\sigma \right)$. The phase difference increment ΔΦ = ΔФ_d_ + ΔФ_g_ = 2*l*_2_*π*/*N*, with *l*_2_ = *m* + 2*σ* = 3, and the lattice field assumes the topology of a honeycomb lattice. Again, while the illuminating light shifts to RCP with *σ* = −1, the RCP wavefield $E_{\text{PF}}^{-\pi /4}\left(\rho \text{, }\beta ;\sigma \right)$ of the primary spin component will hold the same topology as the hexagonal vortex lattice, with lattice order *l*_2_ = 1 as its counterpart under LCP incidence. However, the LCP wavefield $E_{\text{PF}}^{\pi /4}\left(\rho \text{, }\beta ;\sigma \right)$ of the converted spin component changes its lattice order to *l*_1_ = *m* + 2*σ* = −1, appearing as the topology of a hexagonal vortex lattice, but with an opposite vortex topological charge. Here, we especially notice that the lattice topology of the converted component field under the shift of incident light from LCP to RCP, i.e., under the change in incident helicity from *σ* = 1 to *σ* = −1, changes from a honeycomb lattice of *l*_2_ = 3 to a hexagonal vortex lattice of *l*_1_ = −1. This demonstrates that the metasurface combining both dynamic and geometrical phases enable asymmetric SOI. The detailed characteristics of the lattice fields produced by samples A and B are summarized in Table 1.

**Table 1:** Parameters of samples A and B, denoting a metasurface with *m* = 0 and *m* = 1, respectively.

**Samples**	**A**	**A**	**B**	**B**

Geometric order of spiral *m*	0	0	1	1
Incident spin state *σ*	1	−1	1	−1
Lattice order *l*_1_	0	−2	1	−1
Lattice order *l*_2_	2	0	3	1
Phase increment *l*_1_2*π*/*N*	0	−2*π*/3	*π*/3	−*π*/3
Phase increment *l*_2_2*π*/*N*	2*π*/3	0	*π*	*π*/3

In addition, from the above analysis, we can see that the method may also be extended to design metasurfaces to generate more complicated structured and arrayed light fields, which are of greater significance. When the metasurfaces are used to generate more general light field, different complicated patterns can be generated in the two channels of the primary and the converted spin components, respectively, under certain circularly polarized light illumination; but when illumination is changed to other circularly polarized light, the opposite spin dependence of the geometric phases can result in limitations in the independent and arbitrary generation of the pattern of the converted spin component due to the asymmetric SOI [57, 60]. However, more sophisticated design of propagation phase and geometric phase profiles might overcome such limitations.

## 3 Simulation results and discussion

**Figure 2:**
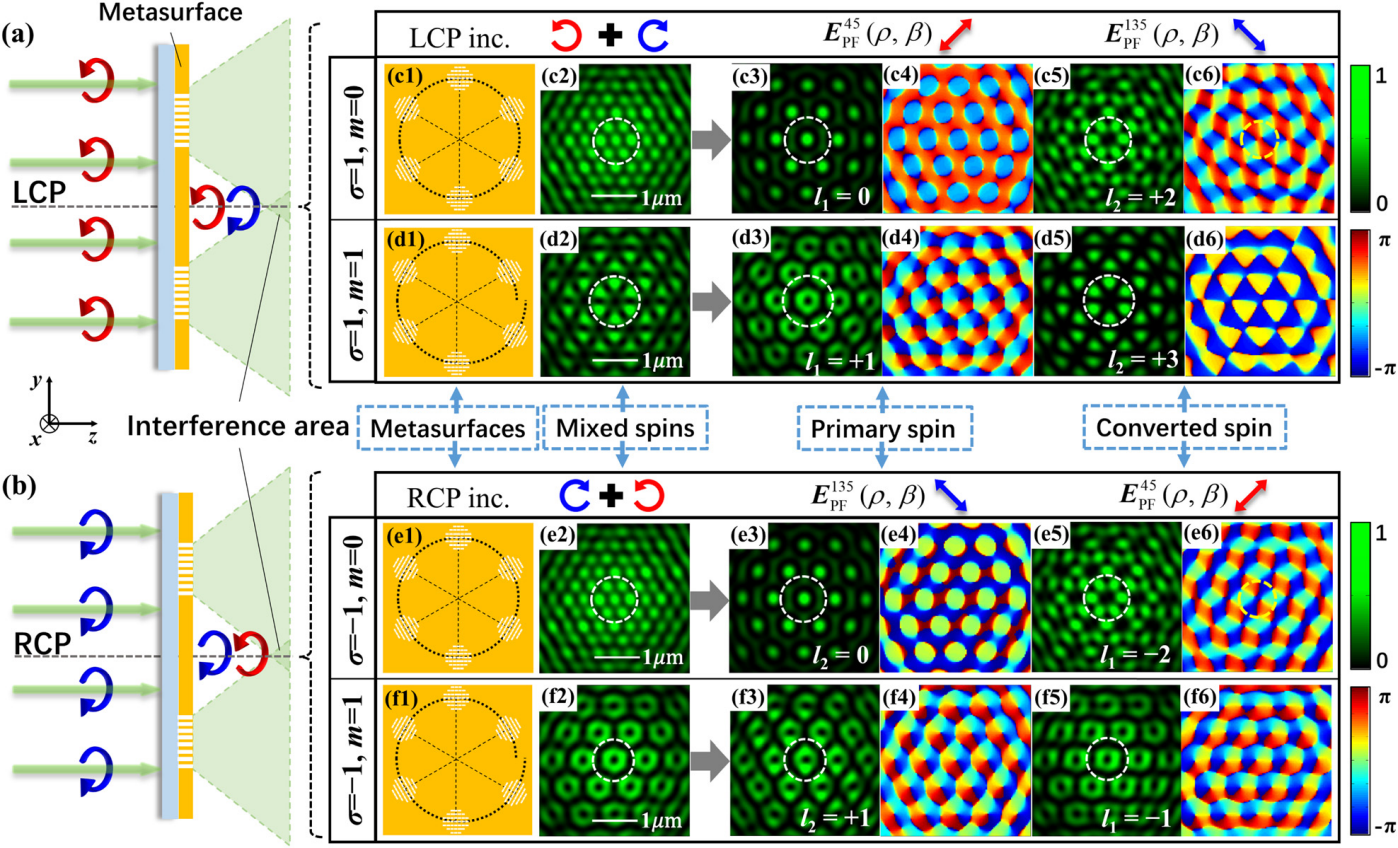
Simulation results of the dual-spin channel lattices induced by metasurfaces of samples A and B: (a) and (b) schematic of the generation of the overlapping lattice fields under the incidence of LCP and RCP, respectively. (c1) and (e1) Sketch map of sample A. (d1) and (f1) Sketch map of sample B. (c2–c6) and (d2–d6) Simulated results of samples A and B, respectively, illuminated by LCP light. (e2–e6) and (f2–f6) Simulated results of samples A and B, respectively, illuminated by RCP light.

To demonstrate the generation of the dual-spin-channel lattices using symmetric and asymmetric SOI, we numerically simulated the lattice wavefields of samples A and B with the aperture centers distributed in a circle and spiral, respectively. The parameters of samples A and B are given in Table 1, and the simulations were implemented using a commercial finite-difference time-domain (FDTD) software (Lumerical Solutions). The simulation area was 30 μm × 30 μm × 4 μm, and the size of the Yee cell was 4 nm. Figure 2(a) and (b) schematically show light of LCP and RCP, respectively, with a wavelength of 532 nm irradiated on the metasurfaces from the substrate side; the wavelength of the SPP waves is *λ*_spp_ = 470 nm he overlapping dual-spin-channel lattice intensities of the primary and converted spin components can be observed in the interference areas at *z* = 1.5 μm on the air side.

Figure 2(c1) and (e1) depict the schematic maps of metasurface sample A, and Figure 2(d1) and (f1) show the map of sample B, with the incidence of LCP and RCP, respectively. We first consider the results for sample A with the symmetric SOI. Under LCP illumination, the parameters are *m* = 0 and *σ* = 1, and the results are shown in Figure 2(c2–c6). Figure 2(c2) shows the unfiltered intensity pattern of a triangle lattice. Then, the polarization filtering operation is numerically simulated to extract the wavefields of the primary and converted spin components from the FDTD data, achieving the wavefields of $E_{\text{PF}}^{\pi /4}\left(\rho \text{, }\beta ;\sigma \right)$ and $E_{\text{PF}}^{-\pi /4}\left(\rho \text{, }\beta ;\sigma \right)$, respectively. Figure 2(c3) is the intensity pattern of the primary spin component numerically filtered as $E_{\text{PF}}^{\pi /4}\left(\rho \text{, }\beta ;\sigma \right)$, denoted in Eq. (13), which is the familiar pattern of a hexagonal lattice with topology *l*_1_ = 0. In the figure, the bright spot surrounded by a dashed circle in white in the lattice center reveals that each beam from the *j*th aperture carries an identical phase, i.e., the phase increment ΔΦ = *l*_1_2*π*/*N* = 0, as shown in Table 1. This leads to an approximately constant phase at bright spots in the phase map, shown in Figure 2(c4). The intensity and phase maps of the converted spin component, numerically filtered as $E_{\text{PF}}^{-\pi /4}\left(\rho \text{, }\beta ;\sigma \right)$, are shown in Figure 2(c5) and (c6), respectively. From Eq. (14) and the discussions in the previous section, the component field has a topology of *l*_2_ = 2, with a phase increment of ΔΦ = *l*_2_2*π*/*N* = 2*π*/3. This represents the Kagome lattice with the cell comprising six elongated lobes, as indicated by a white dashed circle in Figure 2(c5). In the phase map in Figure 2(c6), it can be observed that integrating the phase around a circle marked in yellow dots enclosing the singularity point of dark intensity at the center of the lattice cell will produce a cumulative phase of 2*l*_2_*π* = 4*π*, indicating an optical vortex of order *l*_2_ = 2. Different from the pure orbital angular momentum state in which the phase varies uniformly around the vortex core, the vortex order here refers to the integral topological charge [61] that can be used to describe the phase singularities or phase vortices with nonuniform variation of phases. Undesirably, in the calculated and simulated phase profiles for lattice field of *l*_2_ = 2 in the figures, the patterns appear to be split into two vortices of the same sign, as what is common for a practical vortex of high order to split into several vortices of order 1 [62]. Here we note that *l*_1_ (or *l*_2_) taking integers 0, 1, 2, or 3 signifies the order and the topology of the lattice field, but the first and second order lattice fields contains the phase vortices with the first order and the second order, respectively.

Under RCP illumination, the incident helicity was reversed to *σ* = −1. The unfiltered intensity pattern of the overlapped spin components is shown in Figure 2(e2); it still appears to be a triangle lattice. The intensity pattern of the primary spin component, which is the RCP field of $E_{\text{PF}}^{-\pi /4}\left(\rho \text{, }\beta ;\sigma \right)$, is shown in Figure 2(e3), and has the topology of a hexagonal lattice of the order *l*_2_ = 0, which is the same as that of the primary component under LCP illumination. Figure 2(e4) is the phase map of the component, which has a constant phase difference with its counterpart of LCP illumination. The intensity pattern and phase front map of the converted component, i.e., the LCP field of $E_{\text{PF}}^{-\pi /4}\left(\rho \text{, }\beta ;\sigma \right)$, are shown in Figure 2(e5) and (e6), respectively. We can observe that the intensity pattern still has the topology of a Kagome lattice, which is the same as that in Figure 2(c5) of the converted component under LCP illumination. However, the topology is charged as *l*_1_ = −2 with a phase increment of ΔΦ = *l*_1_2*π*/*N* = −2*π*/3. This manifests as the phase helicity of the wavefront in Figure 2(e6) being opposite to that in Figure 2(c6) for LCP incidence. The characteristic of the wavefield created by the metasurface with symmetric SOI is demonstrated when the intensity distribution remains unchanged with a change in the incident spin helicity. It is also demonstrated that the reversed phase front leads to opposite topological charge when an optical vortex exists in the wavefield.

We now consider the results of sample B with an asymmetric SOI. For the case of the sample under illumination of LCP light, with parameters *m* = 1 and *σ* = 1, the results are shown in Figure 2(d2−d6). Therein, Figure 2(d2) shows the unfiltered intensity pattern with the primary and converted spin components overlapped. Figure 2(d3) is the intensity pattern of the primary spin component, which is the LCP field of $E_{\text{PF}}^{-\pi /4}\left(\rho \text{, }\beta ;\sigma \right)$. It presents the topology of a hexagonal vortex lattice with *l*_1_ = 1, resulting from the dynamic phase difference ΔΦ = *l*_1_2*π*/*N* = *π*/3, as depicted in Table 1. The single vortex constituting the lattice is labeled as a white dashed circle in the figure, and its topological charge is *l*_1_ = 1. This is also demonstrated by the phase map of the primary component field, shown in Figure 2(d4), in which the phase varies over 2*π* on an enclosed path around a vortex core. The intensity pattern of the converted component as the field of $E_{\text{PF}}^{-\pi /4}\left(\rho \text{, }\beta ;\sigma \right)$ is shown in Figure 2(d5), which has the topology of a honeycomb lattice with *l*_2_ = 3 and appears as six hexagon-like lobes. The phase difference ΔΦ = *l*_2_2*π*/*N* = *π* between the beams from two adjacent apertures results in an alternate azimuthal binary phase variation, from −*π* to 0, around the lattice center. Such phase variation indicates the single-phase vortex or optical singularity of topological charge 3 is not formed in the lattice field of *l*_2_ = 3, in contrast to the fields of orders *l*_2_ = 1 and *l*_2_ = 2.

RCP light is used to illuminate sample B, with *m* = 1 and *σ* = −1, and the overlapped intensity pattern of the primary and converted components is shown in Figure 2(f2); interestingly, it depicts the pattern of a hexagonal vortex lattice. The lattice of the primary spin component is the RCP field of $E_{\text{PF}}^{-\pi /4}\left(\rho \text{, }\beta ;\sigma \right)$, and its intensity pattern and phase map are shown in Figure 2(f3) and (f4), respectively. They present the same optical vortex array of the lattice with *l*_2_ = 1 as their correspondents in Figure 2(d3) and (d4) under the LCP illumination, except for the constant phase difference of *π*/2 in the RCP and LCP fields. The intensity and phase maps of the converted spin component are shown in Figure 2(f5) and (f6), and it is the LCP field of $E_{\text{PF}}^{-\pi /4}\left(\rho \text{, }\beta ;\sigma \right)$. The topology of the hexagonal vortex lattice and the clockwise increase of the phase indicate a lattice order of *l*_2_ = −1 with a topological charge of −1 of the optical vortices. The results for sample B indicate the topology transition of the converted component from a honeycomb to a hexagonal vortex lattice, and the unchanged hexagonal vortex lattice topology of the primary component, with the illuminating light shifting from LCP to RCP. This demonstrates that by introducing polarization filtering schemes, metasurfaces of asymmetric SOI realize the manipulation of two converted component channels of $E_{\text{PF}}^{-\pi /4}\left(\rho \text{, }\beta ;\sigma \right)$ and $E_{\text{PF}}^{\pi /4}\left(\rho \text{, }\beta ;\sigma \right)$ by combining the dynamic and the geometric phases. Additionally, they enable the control of the primary spin components to achieve the required lattice field, instead of disregarding it to be scattered into the background.

## 4 Experimental results

**Figure 3:**
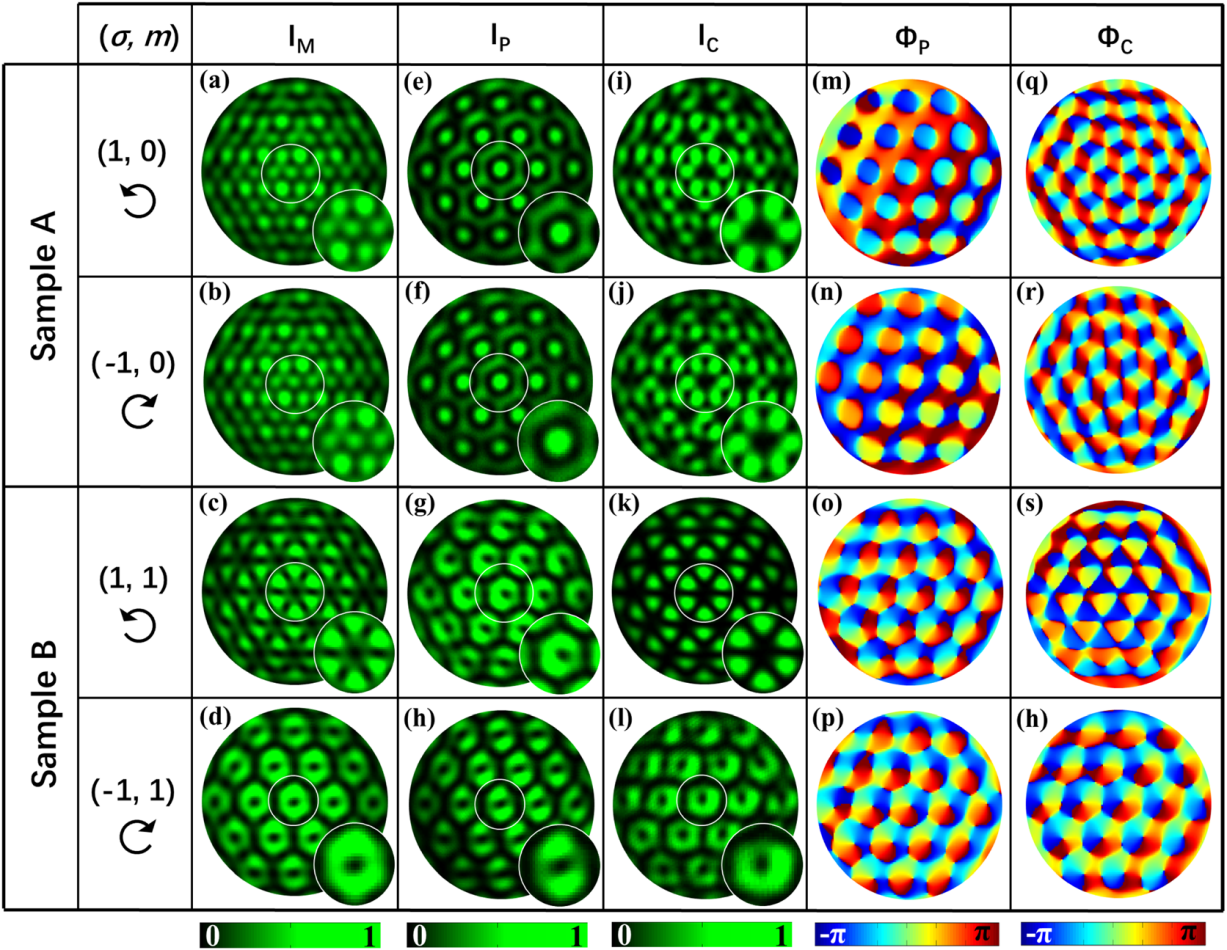
Experimental results for samples A and B under illumination of LCP and RCP light: (a)–(d) Measured intensity patterns for the overlapping lattices of the mixed spin components (*I*_M_). (e)–(h) and (i)–(l) Measured intensity distributions for the primary spin components (*I*_P_) and converted spin components (*I*_C_), respectively, and the enlarged views of the center lattice unit, labeled in white circles, are shown in the insets. (m)–(p) and (q)–(h) Experimental phase maps (Φ_P_ and Φ_C_) corresponding to the two spin components. All the black open circle arrows represent the polarization of the incident light.

Figure 3 shows the experimental results of the lattice patterns for samples A and B under LCP and RCP illuminations, where Figure 3(a–d) show the intensity patterns of the overlapping lattices without polarization filtering; Figure 3(e–h) and Figure 3(i–l) depict the intensity patterns of the primary and converted spin components, respectively. Each inset shows the corresponding magnified view of the central lattice unit. These results show that all lattice fields are well generated. Figure 3(m–p) and Figure 3(q–t) present the phase distributions of the primary and converted spin components, respectively. From Figure 3(e) and (f) and Figure 3(i) and (j), the intensities for sample A with an incidence of LCP and RCP are shown as the changeless hexagonal and Kagome lattices for the primary and converted spin components, respectively, demonstrating the symmetric SOI of the sample. The topologies of 0 and ±2 can be validated by the phase maps in Figure 3(m), (n) and (q), (r), respectively; i.e., identical phase distributions for the two hexagonal lattices of the primary spin components and opposite phase distributions for the two Kagome lattices of the converted spin components, except for the phase difference of *π*/2 between the two components with opposite spin states. Figure 3(g) and (h) display the intensity patterns of the hexagonal vortex lattice in the primary spin channel for sample B with an incidence of LCP and RCP, respectively, indicating spin-independent hexagonal vortex lattice topology of an order of 1. Figure 3(k) and (l) depict the honeycomb lattice and hexagonal vortex lattice of the converted spin component for the sample, indicating spin-dependent topologies of +3 and −1. Though on the whole, the experimental results validate the conclusions drawn from the above theoretical analysis and simulations, we also notice that there are some discrepancies between the experimental results in Figure 3 and the simulated results in Figure 2. These differences are mainly due to the unavoidable factors which may deteriorate the quality of the lattice field in the experimental performances, such as the errors of nanoslit sizes in fabricating metasurfaces, and the imperfectness of optical elements, and misalignment in the optical setup for the lattice field measurement.

## 5 Methods

**Figure 4:**
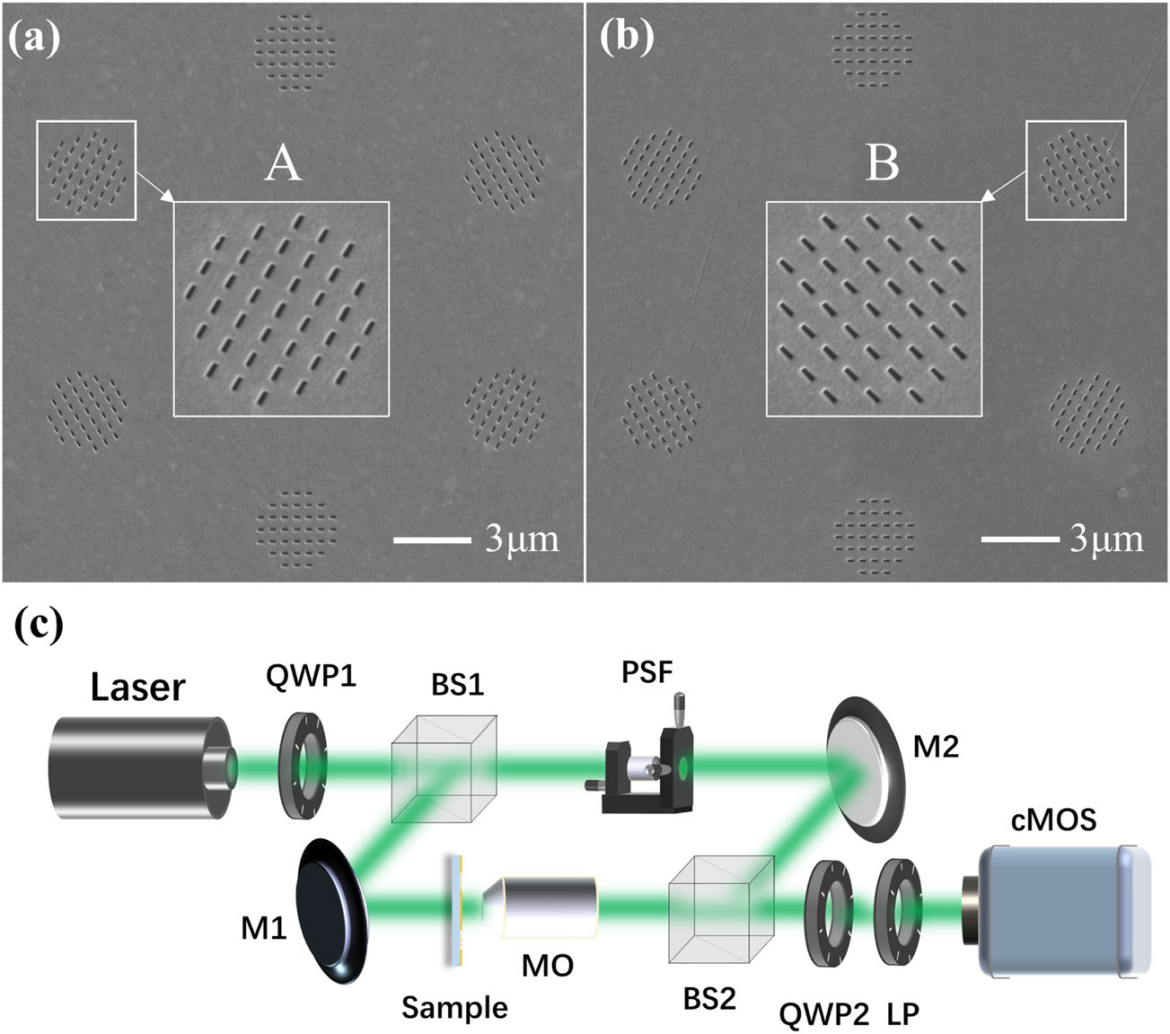
SEM images for samples and experimental setup: (a) and (b) show the SEM images of samples A and B, respectively. (c) Schematic diagram of the experimental setup.

Dual-spin-channel lattices were observed in experiments with the two metasurface samples perforated in a gold film using focused ion beam milling. The gold film was deposited on a glass substrate of 500 μm thickness. Figure 4(a) and (b) shows the scanning electron microscopy (SEM) images of samples A and B, the parameters of which are shown in Table 1. Figure 4(c) shows a schematic of the experimental setup of a Mach–Zehnder interferometer consisting of two beam splitters (BS1 and BS2) and two planar mirrors (M1 and M2). A laser, at a wavelength of 532 nm, and QWP (QWP1) were utilized to obtain circular polarized light to illuminate the sample from its substrate side. The intensity patterns of the overlapping lattices at the position *Z* = 1.5 μm behind the metasurface were magnified by a microscope objective lens (MO, numerical aperture = 0.9/100×) and then captured by a scientific complementary metal–oxide–semiconductor (s-CMOS) camera (Zyla-5.5, Andor, 16 bit). The PF, composed of a QWP (QWP2) and LP were placed before the s-CMOS to filter the primary or converted spin component from the overlapping lattices. A uniform spherical wave, serving as a reference beam, was obtained using a pinhole spatial filter (PSF), with which the beam of the lattice interfered. The intensity and interference patterns were recorded by the s-CMOS camera, and the phase distributions of the imaged lattices were reconstructed from the interference patterns.

## 6 Discussions and conclusions

The present metasurface designs have concentrated on the normal incidence of the illuminating light. When the incident angle of the illuminating light is changed to *θ*_inc_, the additional phase factor exp[*ik*_sp_*x* sin *θ*_inc_] is imposed on the incident light, and it will cause asymmetric spatial changes in the propagation phase differences for light waves of the six holes, and resultantly the phase differences of light fields are mismatched for the generation of designed lattices. This means that the designed metasurfaces are angle-dependent. However, when the wavelength of illuminating light is changed under normal incidence, the nanoslits with the symmetrical spatial arrangement in the apertures will correspond to the optical path changes in proportion for changed wavelength due to the phase factor $\text{exp}\left[i2\pi k_{\text{sp}}\cdot l/\lambda_{\text{sp}}\right]=\text{exp}\left[i2\pi {\hat{k}}_{\text{sp}}\cdot l/\lambda_{\text{sp}}\right]$ with ${\hat{k}}_{\text{sp}}$ being the unit vector, and the propagation phase changes can be generally compensated by adjusting the observation distance; considering the geometric phase being independent of wavelength, the designed metasurfaces possess the broadband characteristics. Our FDTD simulations show that by properly adjusting the observation distance, lattice fields can be obtained in the wavelength range from 472 to 632.8 nm. The efficiency of the designed metasurfaces which is defined as ratio of the power of light forming the lattice field in the far field to that of the incident light illuminating the aperture is calculated by the FDTD simulations, and it is 1.54, 0.79 and 0.70%, respectively, for wavelengths 472 , 532 and 632.8 nm. The low efficiency is mainly resulted from the low aspect ratio of the nanoslits in the metasurfaces.

For further discussions, here we mention several classical designs of metasurfaces related to the combination of dynamic and geometric phases. In the earlier work, Xiao et al. [63] performed the independent manipulations of the plasmonic fields matching the two spin components converted from the illuminating lights by controlling the constructive interference to form the target fields. In the work by Mueller et al. [28], the adjustable dimensions of the dielectric nanopillars in the metasurfaces were designed to convert the chirality of two orthogonal polarization states with dynamic phase imposed simultaneously, and the dynamic and geometric phases was combined to control the light fields of the two converted polarizations, with two images achieved at the polarization channels. As a parallelism in terahertz regimes to the above work, the delicate metasurfaces with meta-atoms capable of converting chirality of the circular polarization and imposing dynamic phase were designed recently by Wang et al. [64], and the manipulations of the spoof surface plasmons corresponding to either converted circular polarizations were demonstrated by beam deflection and focusing. Compared to studies in the literature, the present work makes use of dynamic phase that is related to the optical path, rather than dynamic phase related to the dimension of the nanostructures of the metasurfaces. The functionality of a nanoslit as linear polarizer is directly used to generate the different lattice fields in the two spin channels and under illuminations of the two different circular polarizations. In contrast to the two images of the converted spin fields achieved in most of the literature, the present work has made use of the asymmetric SOI, and achieved another lattice field of either primary spin in addition to the two lattice fields of converted spins.

In summary, based on the SOI effect, the primary and converted spin components transformed from circularly polarized light are used as two spin channels for independent manipulations of desired light fields. The converted component is used extensively for light field manipulations, and its unusual and novel properties have been studied intensely; however, metasurfaces are becoming a novel platform of unprecedented convenience and flexibility for miniatured and planar element designs. The primary spin field, which is well controlled by the dynamic phase as the light field propagating in ordinary diffractions, has been overlooked to some degree. Moreover, it is often considered as a futile background signal, and some effort has been made to eliminate it in recent studies [65]. The present study has demonstrated the manipulation of the two spin component fields in a single-layer plasmonic metasurface. By mimicking multi-beam interference using rotational nanoslit arrays arranged in six round apertures, different hexagon-based lattices are generated in the two component channels. The lattice topologies are controlled by cooperatively modulating the PB phase 2*σθ*_*j*_ and dynamic phase *mθ*_*j*_ associated with the rotation of the nanoslits and the location of the apertures on the spiral, respectively. Particularly, the lattice topologies of the primary spin component can be easily regulated by adjusting the spiral pitch, whereas the lattice topologies of the converted spin component can be determined by the rotation of the slits and spiral pitch. Moreover, they may be symmetrically or asymmetrically helicity-dependent. In practice, spatially overlapped lattice fields of the two spin components are separated by a PF. Notably, the primary spin component has been unintentionally used in some previous studies, such as vector beam generation with metasurfaces, where the fields of the primary and converted components are vectorially superposed without being independently manipulated [48]. We intend for the present study to provide more flexible and efficient manipulations on light fields and allow for micro-manipulations, topological photonics, and on-chip quantum optical applications. Particularly, we hope that our design to be generalized to the Moiré metasurfaces [66, 67] for the dynamic spin manipulations through the rotations of metasurface layers, which has become one of the most important fields in recent metasurface applications, and in addition, such generalization for the Moiré patterns of honeycomb lattices might be related to the magic angle effect in graphene which has been paid attention to in topological photonics recently.
